# Reproducibility of Aquablation outcomes across surgeon experience levels: A descriptive analysis of published studies

**DOI:** 10.1002/bco2.70282

**Published:** 2026-09-20

**Authors:** Eviatar Fields, Mario Henrique Bitar Siqueira, Philip Charlesworth, Garrett Pohlman, David Cuellar, Shin Koike, Naeem Bhojani, Bilal Chughtai, Kevin C. Zorn, Dean Elterman

**Affiliations:** ^1^ Faculty of Medicine McGill University Montréal Canada; ^2^ Division of Urology, Department of Surgery University of Toronto Toronto Canada; ^3^ The Royal Marsden Hospital London UK; ^4^ Kearney Urology Center PC Kearney Nebraska USA; ^5^ Urology Austin PLLC Austin Texas USA; ^6^ Itabashi Chuo Medical Center Tokyo Japan; ^7^ Division of Urology Centre Hospitalier de l'Université de Montréal (CHUM) Montréal Canada; ^8^ Plainview Hospital, Smith Institute of Urology Northwell Health Syosset New York USA; ^9^ Prostate Surgical Institute BPH Canada Montreal Canada

**Keywords:** Aquablation, benign prostatic hyperplasia, BPH, reproducibility, surgeon experience

## Abstract

**Objective:**

The objective of this study is to descriptively evaluate the reported functional and symptomatic outcomes of Aquablation therapy across published series categorized by operator experience level.

**Subjects/Patients and Methods:**

We performed a non‐systematic descriptive review of functional and symptomatic outcomes of eight published Aquablation clinical trials and real‐world series. Studies were categorized based on inferred or known operator experience into early‐experience (Water, Water II and Japan‐PMS; total *n* = 332) and experienced groups (Water III, ASC Pilot, ASC Global, Mount Sinai and Madrid; total *n* = 664). Extracted variables included baseline and follow‐up International Prostate Symptom Score (IPSS), IPSS‐quality of life (QoL), maximum urinary flow rate (Qmax) and post‐void residual volume (PVR). Weighted and sample‐size weighted mean and standard deviation of change were calculated and evaluated descriptively.

**Results:**

IPSS, IPSS‐QoL, Qmax and PVR all improved across the eight cohorts. Mean changes in patient‐reported outcomes were similar between early‐experience and experienced cohorts for IPSS (unweighted mean change: −14.77 vs. −15.15; weighted: −14.81 vs. −15.30) and IPSS‐QoL (unweighted: −3.13 vs. −3.28; weighted: −3.14 vs. −3.13). Physiologic metrics (QMax and PVR) demonstrated higher absolute improvements and greater variance in the experienced cohort, heavily driven by cohorts with more severe baseline urinary retention.

**Conclusions:**

Across published series, Aquablation yields broadly consistent improvements in patient‐reported symptom scores, regardless of baseline operator experience level. Observed differences in physiological outcomes appear primarily influenced by baseline patient selection rather than surgeon experience. These descriptive findings support the general reproducibility of Aquablation outcomes across real‐world clinical settings. Formal prospective studies are required to define reproducibility across perioperative safety profiles.

## 1 INTRODUCTION

Benign prostatic hyperplasia (BPH) is a common, progressive condition causing prostate enlargement in aging men.^1^ It is common, affecting almost 50% of men over age 50 and up to 80% of men greater than 80 years old.^2^ Enlarged prostate may lead to bladder outlet obstruction (BOO), which clinically manifests as lower urinary tract symptoms (LUTS). Indeed, approximately 40% of men with BPH will experience symptoms of LUTS, significantly impacting quality of life.^3^ Given its prevalence, BPH is also a significant burden on the healthcare system.^4^, ^5^ Conservative treatment for BPH includes watchful waiting and pharmaceutical management; however, if prostate size is too large and causes significant LUTS, surgical management is preferred.^6^

Approximately 30% of men with prostate enlargement will require surgery. Numerous endoscopic treatments for BPH exist with various benefits and levels of invasiveness, including transurethral resection of the prostate (TURP), holmium laser enucleation of the prostate (HoLEP), GreenLight™ photoselective vaporization of the prostate (PVP), Aquablation, UroLift and Rezūm.^5^, ^7^, ^8^ Of these, Aquablation has recently proven to have durable beneficial functional and quality of life outcomes.^3^, ^9^, ^10^ Aquablation utilizes the AQUABEAM Robotic System, or more recently the HYDROS Robotic System, to perform highly accurate, ultrasound‐guided, robotic‐assisted hydro‐dissection of the prostate.^9^, ^11^ Clinical trials have demonstrated sustained efficacy of Aquablation for a wide range of prostates^10^, ^12^ up to 5 years post‐operatively,^13^ as well as reduced incontinence, erectile and ejaculatory side effects^14^, ^15^ compared to other techniques.^16^ Importantly, Aquablation has a favourable safety profile,^10^, ^12^, ^14^ and its safety and efficacy have been demonstrated in numerous real‐world and post‐marketing studies.^10^, ^17^, ^18^, ^19^

Aquablation was initially approved by the FDA in 2017,^11^ with the WATER trial being published the following year.^20^ As with all novel therapies, reproducibility across centres is essential for validating the safety and efficacy of new surgical technologies. One factor that can significantly impact reproducibility is surgeon experience with a technique, which has been shown to impact outcomes of other BPH therapies.^21^, ^22^ Although Aquablation shows positive outcomes in global clinical trials across North America, Asia and Europe, few studies have examined how surgeon experience may affect its outcomes. As adoption of Aquablation expands globally beyond high‐volume academic centres, understanding whether outcomes remain consistent across varying surgeon experience is critical for guiding patient care.^10^, ^17^

Here, we perform a descriptive analysis of aggregate data from eight published Aquablation trials and real‐world series from centres with varying procedural expertise, to better examine the reproducibility of functional and symptomatic outcomes of Aquablation therapy.

## 2 METHODS

A non‐systematic, descriptive analysis was performed comparing eight major Aquablation studies, stratified by surgeon experience. Studies were not selected systematically, rather based on inferred experience level of surgeons. Experience was inferred by initial recruitment date for a study or stated experience of the surgeon (Table S1). The early‐experience group included Hinata et al. (Japan‐PMS),^18^ WATER^13^ and WATER II,^13^ which were conducted by surgeons with stated no prior exposure to Aquablation or prior to 2017. The experienced group included WATER III,^23^ Marhamati et al. (ASC Pilot),^24^ Zorn et al. (ASC Global),^17^ Omidele et al. (Mount Sinai),^25^ and Justo Quintas et al. (Madrid),^16^ which were conducted by surgeons with stated prior experience with Aquablation or after 2021.

Extracted variables included baseline and follow‐up International Prostate Symptom Score (IPSS), IPSS Quality of Life (IPSS‐QoL), maximum urinary flow rate (Qmax) and post‐void residual volume (PVR), as well as standard deviation (SD) for all values. If SD was unavailable (i.e., ASC Pilot and Global^17^, ^24^), it was inferred using the available published ranges for that study, based on the Range Rule of Thumb:

$$\text{For small sample size}\;\left(n<70\right):\mathrm{SD}\approx \frac{\text{Range}}{4}.$$

For large sample size (*n* > 70), $\mathrm{SD}\approx \frac{\text{Range}}{6}$.

One study (Water II) did not report SD or numerical ranges,^13^ so relevant plots were digitized, and SD was inferred based on resulting range. Using this information, mean change and SD of change for IPSS, IPSS‐QoL Qmax and PVR were calculated for each study. Both unweighted and sample‐size‐weighted group means were reported. The standard Cochrane formula for calculating *SD*_*change*_ was used as follows, with the correlation coefficient set at *r* = 0.5:

$${\mathrm{SD}}_{\text{change}}=\sqrt{{\mathrm{SD}}_{\text{baseline}}^{2}+{\mathrm{SD}}_{\text{follow}-\mathrm{up}}^{2}-\left(2\cdot r\cdot {\mathrm{SD}}_{\text{baseline}}\cdot {\mathrm{SD}}_{\text{follow}-\mathrm{up}}\right)}.$$

Due to heterogeneity of the available data, we did not perform patient‐level pooled analysis, a meta‐analysis or random‐effects weighting. Data were analysed descriptively, and no statistical analyses were performed.

This manuscript contains only published aggregated data. As we did not include any new patient data, we did not require ethics approval. All original studies had appropriate ethics approval.

## 3 RESULTS

### 3.1 Patient demographics

We included follow‐up data from eight Aquablation studies in our descriptive analysis.^13^, ^16^, ^17^, ^18^, ^24^, ^25^ The early‐experience group consisted of three trials including Japan‐PMS (*n* = 103),^18^ WATER (*n* = 116)^13^ and WATER II (*n* = 103)^13^ studies, whereas the experienced group consisted of five trials, including WATER III^23^ (*n* = 98), ASC Pilot (*n* = 101),^24^ ASC Global (*n* = 60),^17^ Mount Sinai (*n* = 330)^25^ and Madrid (*n* = 75)^16^ studies. In total, there were 322 patients in the early‐experience trials and 664 patients in the experienced trials, for a total of 986 patients across all trials. The surgeons in the early‐experience trials had no prior use of the AQUABEAM system (Table S1). The studies represented their first cases in Aquablation. The experienced group of studies was among surgeons who had already been using the AQUABEAM system in prior clinical trials and in clinical practice (Table S1).

Follow‐up time varied drastically throughout the trials, with the early‐experience group ranging from 6 to 60 months while the experienced group ranged from 1 to 48 months. Age within the early‐experience group ranged from 65.9–71.1 and 65.2–68.6 years within the experienced group. Both groups included patients with prostates ranging from small (less than 30 ml) to very‐large (over 150 ml), and the mean prostate size ranged from 54.1 to 107.4 ml in the early‐experience group and 66.3 to 108.4 ml in the experienced group.

### 3.2 Functional outcomes

#### 3.2.1 IPSS

Baseline IPSS values within the early‐experience group ranged from 18.10 (9.00) to 23.20 (6.30) in Japan and WATER II, respectively. In the experienced group, the range of baseline values was larger, from 20.20 (7.10) to 29.70 (4.50) in Madrid and ASC Global respectively. Generally, all eight trials improved in IPSS.

We assessed mean change in IPSS between the early‐experience and experienced groups (Table 1). Mean changes in IPSS within the early‐experience groups were generally similar, ranging from −12.00 in Japan‐PMS to −16.40 in WATER II. In the experienced group, the mean change in IPSS was more variable, ranging from −11.60 in WATER III to −23.10 in ASC Global. Weighted group mean changes in the early‐experience and experienced groups were −14.81 and −15.30, respectively. Like with mean change, SD of change was similar across the three early‐experience groups (range: 6.39 to 7.81) and variable within the experienced group (range: 3.91 to 7.40).

**TABLE 1:** IPSS and IPSS‐QoL values for the various studies included.

	IPSS				
	Study	Baseline (SD)	Follow‐up (SD)	Mean change	SD change
Early‐experience	Japan‐PMS	18.10 (9.00)	6.10 (5.00)	−12.00	7.81
	Water I	22.90 (6.00)	7.00 (8.52)	−15.90	7.58
	Water II^a^	23.20 (6.30)	6.80 (6.48)	−16.40	6.39
	Group change	−14.77
	Group change (weighted)	−14.81
	Mount Sinai	23.80 (8.40)	6.90 (2.90)	−16.90	7.39
	Madrid	20.20 (7.10)	5.05 (4.90)	−15.15	6.30
	ASC Global^b^	29.70 (4.50)	6.60 (2.00)	−23.10	3.91
Experienced	ASC Pilot^b^	22.00 (4.67)	9.20 (4.33)	−12.80	4.51
	Water III	20.70 (5.70)	12.9 (6.9)	−7.80	6.39
	Group change (unweighted)	−15.15			
	Group change (weighted)	−15.30			
	IPSS‐QoL				
Early‐experience	Japan‐PMS	4.90 (1.30)	2.20 (1.80)	−2.70	1.61
	Water I	4.80 (1.10)	1.60 (1.65)	−3.20	1.45
	Water II	4.60 (1.00)	1.10 (1.29)	−3.50	1.18
	Group change (unweighted)	−3.13			
	Group change (Weighted)	−3.14			
Experienced	Mount Sinai	–	–	–	–
	Madrid	4.35 (1.41)	1.30 (1.60)	−3.05	1.51
	ASC Global	5.20 (0.75)	0.90 (0.50)	−4.30	0.66
	ASC Pilot	4.40 (0.83)	1.90 (1.00)	−2.50	0.93
	Water III	4.20 (1.50)	–	–	–
	Group change (unweighted)	−3.28			
	Group change (weighted)	−3.13			

Abbreviations: IPSS, international prostate symptom score; IPSS‐QoL, international prostate symptom score‐quality of life; SD, standard deviation.

^a^ Data for SD were abstracted from digitized published figures.

^b^ Data for SD were imputed using range rule of thumb method (see Section 2).

#### 3.2.2 IPSS quality of life

We extracted available IPSS quality of life (IPSS‐QoL) outcomes from all three early‐experience trials and from three of the five experienced groups (Madrid, ASC Global and ASC Pilot; Table 1). Baseline data were similar for all six groups: The early‐experience group ranged from 4.60 (1.00) in WATER II to 4.90 (1.30) in Japan‐PMS, whereas the experienced group ranged from 4.20 (1.50) in WATER III to 5.20 (0.75) in ASC Global. IPSS‐QoL improved in all six trials with available data. The weighted group mean change was similar across the groups, with a group mean change of −3.14 (range: −2.50, −4.30) in the early‐experience group and −3.13 (range: −2.70, −3.50) in the experienced group.

#### 3.2.3 Qmax

Maximal flowrate (Qmax) in the early‐experience group was within 1.10 ml/s, ranging from 8.30 ml/s (SD: 4.40) in Japan to 9.40 ml/s (SD: 3.30) in WATER (Table 2). In contrast, there was a larger range for Qmax in the experienced group, with the lowest Qmax found in ASC Global (5.90 ml/s [2.00]) and almost double in WATER III (10.70 ml/s [4.50]). Qmax improved consistently following Aquablation in all eight trials.

**TABLE 2:** Qmax and PVR values for the various studies included.

	Qmax (ml/s)				
	Study	Baseline (SD)	Follow‐up (SD)	Mean Change	SD Change
Early experience	Japan‐PMS	8.30 (4.40)	15.50 (7.80)	7.2	6.77
	WATER I	9.40 (3.30)	17.30 (14.29)	7.9	12.96
	WATER II^a^	8.70 (3.62)	17.10 (14.24)	8.4	12.82
	Group change (unweighted)	7.83			
	Group change (weighted)	7.84			
Experienced	Mount Sinai	6.40 (4.20)	17.40 (5.50)	11	4.98
	Madrid	9.05 (3.60)	23.80 (9.30)	14.75	8.12
	ASC‐Global^b^	5.90 (2.00)	26.30 (9.00)	20.4	8.19
	ASC‐Pilot^b^	10.60 (4.17)	18.30 (4.68)	7.7	4.45
	WATER III	10.70 (4.50)	19.80 (10.6)	9.1	9.21
	Group change (unweighted)	12.59			
	Group change (weighted)	11.49			
		PVR (ml)
Early experience	Japan‐PMS	85.60 (107.20)	43.40 (60.00)	−42.20	93.06
	WATER I	97.00 (82.43)	42.00 (96.16)	−55.00	90.08
	WATER II	131.00 (129.45)	64.00 (95.79)	−67.00	116.33
	Group change (unweighted)	−54.73			
	Group change (weighted)	−54.74			
Experienced	Mount Sinai	121.80 (39.70)	31.40 (11.50)	−90.40	35.38
	Madrid	69.80 (48.50)	20.30 (22.60)	−49.50	42.03
	ASC‐Global	408.00 (304.00)	39.00 (30.00)	−369.00	290.17
	ASC‐Pilot	161.00 (101.33)	40.10 (43.33)	−120.90	88.06
	WATER III	101.20 (81.80)	48.00 (66.00)	−53.20	75.16
	Group change (unweighted)	−136.60			
	Group change (weighted)	−110.10			

Abbreviations: PVR, post‐void residual volume; Qmax, maximum flow rate; SD, standard deviation.

^a^ Data for SD were imputed using range rule of thumb method (Section 2).

^b^ Data for SD were abstracted from digitized published figures.

In the early‐experience group, the mean change in Qmax remained similar across all groups, ranging from 7.20 ml/s in Japan‐PMS to 8.40 ml/s in WATER II, with a weighted group mean of 7.84 ml/s. On the other hand, Qmax varied within the experience group, ranging from 7.70 ml/s in ASC Pilot to 20.40 ml/s in ASC Global. The weighted group mean for the experienced group was 11.49 ml/s. SD of change also varied within the groups. In the early‐experience group, SD of change was 6.77 ml/s in Japan‐PMS and 12.96 ml/s in WATER II. Within the experienced group, SD of change was lowest in Mount Sinai at 4.98 ml/s and almost double in ASC Global (8.19 ml/s).

#### 3.2.4 PVR

As with Qmax, there was variability in post‐void residual (PVR) data between the trials (Table 2). In the early‐experience group, baseline data ranged from 85.60 ml (SD: 107.20) to 131.00 ml (SD: 129.45). This variability was more extreme in the experienced group ranging from 69.80 ml (SD: 48.50) in Madrid to 408 ml (SD: 304.00) in ASC Global. All eight trials examined improved in PVR after Aquablation.

In the early‐experience group, improvements in PVR ranged from −42.20 ml in Japan‐PMS to −67.00 ml in WATER II, with a weighted group mean change of −54.74 ml. Improvement in all five experienced groups surpassed the smallest improvement in the early‐experience group (i.e., Japan‐PMS); however, there were three distinct improvement groups within experienced trials: The Madrid and WATER III showed modest improvement (−49.50 and −53.20 ml, respectively), Mount Sinai and ASC Pilot, showed greater improvement (−90.40 and −120.88 ml), and ASC Global demonstrated massive improvement in PVR (−369.00 ml). The weighted mean change in the experience group was −110.10 ml, almost double that of the early‐experience group. There was also spread in SD of change in both groups. The early‐experience group ranged from 93.06 to 116.33 ml, whereas the SD of change in the experience group ranged from 35.38 to 290.17 ml.

## 4 DISCUSSION

Here, we describe IPSS, IPSS‐QoL, Qmax and PVR outcomes following Aquablation in eight trials and stratify results based on experience level of the surgeons performing the procedure. Generally, we observed an improvement in all four outcomes following Aquablation across the eight included studies. Group‐level improvement in all patient‐reported outcomes (i.e., IPSS and IPSS‐QoL) were remarkably similar, despite differences in surgeon experience. In physiological outcomes (Qmax and PVR), we observed a potentially greater improvement in outcomes for experienced surgeons, although there was a larger spread within the data. Although this study remained descriptive and does not perform statistical comparisons, we observed that despite operator experience, Aquablation generally provides robust reproducibility of symptomatic and functional outcomes across centres.

Surgeon experience has long been a known variable impacting patient outcomes.^21^, ^22^, ^26^, ^27^, ^28^ As such, effort has been made to quantify learning curves for many urological procedures.^28^ Various learning curves have been published for prostate resection, suggesting that proficiency is reached after over 100 cases for Greenlight™ laser enucleation of the prostate (LEP)^27^, ^29^ and PVP,^30^ 40–60 cases for HoLEP^28^ and 50 cases for TURP.^31^ Recent work has found that only 38 cases are required for proficiency in Aquablation,^32^ suggesting that Aquablation is no more difficult than other gold‐standard treatments for BPH.

Patient‐reported outcomes were similar between the early‐experience and experienced groups, which may suggest that Aquablation outcomes are less sensitive to learning‐curve effects than other endoscopic BPH procedures. There are a few potential reasons for this. The robotic automation involved in Aquablation, coupled with live TRUS‐guided prostate mapping,^11^ may remove significant operator‐variability that may otherwise lead to increased reliance on surgeon experience in traditional manual techniques such as HoLEP, Greenlight™ LEP/PVP and TURP. Further, a 38‐case learning curve^32^ is relatively low for achieving proficiency. As a result, perhaps the early‐experience group achieved relative mastery of the Aquablation technique by the time they completed the Japan‐PMS, WATER and WATER II studies, which could eliminate between‐group variability in surgeon experience. In this case, there may have only been minimal difference in surgeon experience between the early‐experience and experienced groups, which could account for the remarkably consistent outcomes across sites. This may also account for the higher improvement values and variability in physiological outcomes seen in the experienced group studies, as these surgeons also continue to improve throughout their respective studies. However, the lack of objective measures of surgeon experience reported by our study (e.g., learning curves and case‐loads) prevents us from conclusively determine this, and future studies should strive to objectively compare the reproducibility of Aquablation across learning curves.

It is important to emphasize that we are not claiming that surgeon experience is irrelevant to Aquablation outcomes nor that a learning curve is absent. Rather, our descriptive data suggest that both early‐experience and experienced cohorts achieved clinically meaningful improvements, even with little prior exposure to Aquablation. This is important from a clinical perspective, as it allows for technical democratization of this technique across institutions. Indeed, analysis of real‐world data from the FDA Manufacturer and User Facility Device Experience (MAUDE) database has already demonstrated its safety in over 70 000 patients,^10^ and numerous studies have shown its efficacy in ambulatory care centers.^17^, ^24^ Thus, our hypothesis‐generating observations support the continued adoption of Aquablation in community healthcare centres and outpatient clinics, though this should be confirmed with dedicated prospective studies incorporating surgeon‐level experience data.

We observed large differences in physiologic outcomes (Qmax and PVR) between cohorts. These differences are heavily confounded by baseline symptom severity and thus likely reflect heterogeneity in patient selection rather than surgeon expertise. For example, the higher baseline PVR values observed in the ASC Global cohort permit substantially larger absolute numerical drops in PVR post‐Aquablation. We also noticed an increased variability in physiologic outcomes compared with patient‐reported outcome metrics (PROM; IPSS and IPSS‐QoL), especially within the experienced group. Generally, PROMs are standardized and thus more immune to operator dependency.^30^ In contrast, physiological metrics may be influenced by other values such as operator experience and patient selection.^33^ The ASC Global study in particular saw much greater improvement than the other included studies and may be driving much of the observed improvement in PVR for the experienced group. Given that improvements in both PROM and physiological metrics typically continue to improve for 12 months after surgery,^9^ the 1 month follow‐up period of the ASC Global study suggests that patient selection was a key driver behind the results of this study.

The minimal variability in mean change for PROMs between the early‐experience and experienced groups further demonstrates the benefits of Aquablation, as there is clear patient‐perceived improvement despite variability in uroflow metrics. All eight trials more than doubled the reported minimally clinical important difference (MCID) in IPSS of 5.26,^34^ suggesting that clinically meaningful outcomes are easily achieved, even by novice surgeons. Although there is no standardized MCID for physiological outcomes, improvement in both PROM^34^ and physiologic outcomes^33^ after BPH therapy is known to be influenced by initial severity of symptoms; the more severe the symptoms, the larger the improvement. This could also explain the extreme results of the ASC Global study, which had the most severe baseline scores and largest improvement in all outcomes. Indeed, this likely contributed to the almost threefold difference in PVR improvement delta between the cohorts; removal of this outlier would likely shrink the delta between the two cohorts, further eliminating any difference in outcomes related to surgeon experience.

Our study is not without limitations. First, we did not collect these studies systematically, follow PRISMA guidelines or perform a risk of bias assessment, which could add inherent selection bias to our conclusions as studies were hand‐picked to better stratify by experience. We did not perform any statistical analyses to compare the groups, and all results within this study should be interpreted as purely descriptive observations. Our studies were also not uniform in follow‐up time, ranging from 1 to 60 months, which is known to have a significant effect on outcome measures. Further, we had to infer missing SD data from some studies, and there were some differences among baseline characteristics. For example, the Japan‐PMS study had a notably older population with an average age of 71.1, which may impact outcomes; Raizenne et al.,^35^ for example, compared men over or under 65 years of age undergoing and found that although Rezūm improved outcomes for all men, the improvement of Qmax in elderly men was less pronounced. There is thus likely significant heterogeneity within our results that is not reported. Due to the heterogeneity of the literature, we did not report on safety and perioperative outcomes, which warrant a dedicated comparative investigation in the future. Finally, our categorization of studies into the early‐experience and experienced cohorts was based on known prior exposure to the Aquablation techniques, rather than objective measures such as learning curves. Thus, our lack of objective quantification of surgeon experience may mask nuances within the cohorts themselves. Given these limitations, this study is intended to be interpreted as hypothesis‐generating and supportive of reproducibility, rather than hypothesis‐testing.

## 5 CONCLUSIONS

Both novice and experienced operators achieved substantial improvements in IPSS, Qmax and PVR. Although these descriptive findings suggest a broad reproducibility of Aquablation outcomes across diverse clinical settings, formal prospective studies with individual surgeon‐level data are needed.

## AUTHOR CONTRIBUTIONS

Eviatar Fields and Mario Henrique Bitar Siqueira contributed to the conceptualization of the study and drafted the manuscript. Philip Charlesworth, Garrett Pohlman, David Cuellar, Shin Koike, Naeem Bhojani, Bilal Chughtai and Kevin C. Zorn contributed to data collection, data curation, investigation and critical revision of the manuscript. Dean Elterman supervised the study, contributed to data analysis and interpretation, and critically revised the manuscript. All authors reviewed and approved the final version of the manuscript.

## CONFLICT OF INTEREST STATEMENT

Eviatar Fields and Mario Henrique Bitar Siqueira declare that no conflicts of interest exist. Philip Charlesworth, Garrett Pohlman, David Cuellar, Shin Koike, Naeem Bhojani, Bilal Chughtai, Kevin C. Zorn and Dean Elterman are PROCEPT BioRobotics Investigators.

## Supporting information


**Table S1:** Reasoning for grouping of trials by surgeon experience. Japan [18], and Water I [20] and II [13] made up the early‐experience group, while Water III[23], Mount Sinai[25], Madrid [16], ASC Pilot [24] and ASC Global [17] comprised the experienced group.

## Data Availability

These data did not involve the collection of new patient‐level data, and data were extracted and analysed from previously published reports. Data include baseline and follow‐up values, reported or imputed standards of deviation, sample sizes, follow‐up time point and the original source publication for each study. No additional unpublished data were used. Data are available from the corresponding author upon reasonable request.
