# Supplementary material for: Reproducibility of Aquablation outcomes across surgeon experience levels: A descriptive analysis of published studies
Source: BJUI Compass. 2026 Sep 20;7(9):e70282. doi: 10.1002/bco2.70282 (PMC13590277; doi:10.1002/bco2.70282)
Supplement: Supplementary file 1 [file BCO2-7-e70282-s001.docx]

| **Study** | **Experience** | **Reason** |
| --- | --- | --- |
| Japan | Early-Experience | Naïve surgeons (state no prior Aquablation experience) |
| Water I | Early-Experience | Inclusion dates 11/2015-12/2016 |
| Water II | Early-Experience | Naïve surgeons (state no prior Aquablation experience) |
| Mount Sinai | Experienced | Real-world experience study; Inclusion dates 2019-2023 |
| Madrid | Experienced | Inclusion dates 06/2021-07/2023 |
| ASC Global | Experienced | Stated experienced surgeon |
| ASC Pilot | Experienced | Inclusion dates 01/2024-06/2025 |
| Water III | Experienced | Stated largely experienced surgeons |
|  |  |  |

Table S1: Reasoning for grouping of trials by surgeon experience. Japan [18], and Water I [20] and II [13] made up the early-experience group, while Water III[23], Mount Sinai[25], Madrid [16], ASC Pilot [24] and ASC Global [17] comprised the experienced group.
